# Some Suggestions on the Treatment of Pyorrhea Alveolaris

**Published:** 1899-07

**Authors:** G. Lenox Curtis

**Affiliations:** New York City.


ARTICLE II.

### SOME SUGGESTIONS ON THE TREATMENT OF PYORRHEA ALVEOLARIS.

G. LENOX CURTIS, M. D., NEW YORK CITY.

Read before Alumni Association of Boston Dental College, December 14th, 1898.

In my remarks I shall not attempt to exhaust this subject, which is broader than the present status of knowledge. I, however, make a few suggestions, with the view of drawing out discussion that may lead to widening the field. I shall not think it strange if some of my hearers disagree with me, for I differ somewhat with most authors upon the subject. I think that Dr. Pierce has fallen into the error made by some other writers, who claim that uric acid is the cause instead of the result of rheumatism and gout, of which pyorrhea alveolaris is a symptom, seen only in certain classes of the discrasia. I have found that it is not always safe to adopt some views, especially those found in some books. Not only dentists, but physicians sometimes depend too largely upon literature, for their practice in most oral diseases.

Perhaps all dentists do not have facilities for thorough experimental work, but they certainly deserve credit for the persistent manner in which they have endeavored to cure, by devoting their time almost wholly to the treatment of a single symptom. I believe that very few recognize the importance of general treatment, with the view of eradicating from the system the conditions that aggravate this disagreeable and offensive malady. There are physicians—even those of prominence—who seem to fail to observe one symptom familiar to every dentist—that is, they do not appear to connect the local aspect with a systemic disorder. In both of these professions the lack of general knowledge concerning the intimacy of the systemic with the local (directly or indirectly) is something surprising. The present plan of medical teaching is far from being equal with that known to the educated dentist more familiar with oral diseases. The medical curriculum will not be effective until its students are better educated upon oral diseases. The etiology of Pyorrhea Alveolaris or Loculosis Alveolaris, to my mind, is best presented by Dr. Eugene S. Talbot, who attributed the principal cause to careless dentistry and degeneracy of some of the oral tissues.

The clinical features are best described by Dr. J. N. Farrar in his articles in the *Independent Practitioner*. April and September, 1886.

Among the local causes for which I believe careless dentists are responsible, is extraction of one or more teeth, leaving thereby imperfect antagonism, improper contour fillings, overlapping fillings between the teeth, ill-fitting root bands and plates, ligature, file and emery strip wounds; possibly too hard malleting is a factor; certainly bunglingly made regulating machines, V-shaped spaces between the teeth, whether caused by file or wedge, allowing lodgment of food, salivary calculi and cheesy deposits. Any local condition that leads to degeneracy, or any medicine that causes salivation should not be overlooked in the diagnosis and treatment. I regard rheumatism, gout and syphilis as potent causes of this malady, but, of course, it does not necessarily follow that these affections always accompany pyorrhea alveolaris. I sometimes think that physicians do not always appreciate the importance of retaining the natural teeth, or substituting artificial ones where only one or more teeth are lost. As the treatment of socket diseases more properly belong to the dentist, and many of them do not thoroughly remove the deposits from the teeth, and a less number have a practical knowledge of the best principles in the treatment. I think more surgical and medical education should be taught in dental colleges, and more dental education taught in medical colleges. Most dentists are general practitioners in their calling, attempting all parts, and do not follow exclusively any one branch until they find that they excel in it. Instead of advising their patients to go to a sperialist for treatment of that in which they do not excel, they too often skim over that, making a feint at treating the disease, or, what is more unpardonable, adopt the "do-nothing-plan," and tell their patients this trouble is incurable.

I believe pyorrhea alveolaris can be cured. This I feel I can prove by tabulated cases. Treating this disease, while practiced by dentists, belongs as much to surgery as the reduction of fracture of a bone. Of course, only the deft in skill can properly treat it. Dr. Dunlap truthfully said, "A doctor can be made in five years, but it requires twenty-five years to make a first-class physician."

A clear knowledge of the condition of the patient is an important matter. The surgeon generally tries to learn this, while the dentist too often overlooks it. The restoration of the general health is essentially the cure of pyorrhea alveolaris, but the general health cannot be accomplished so long as this particular disease exists in an aggravated form. The ordinary physicians rarely examine the mouth to ascertain whether there are sufficient teeth to masticate with, and overlook the common fact, that there are often pyorrhea pockets filled with bacteria and pus, daily being mixed with the food, and continually carried into the stomach. Nearly all persons affected with this disease have dyspeptic ailments, anemia, blood poisoning, and accompanying these there may be various forms of neuralgia and occasionally, mania. A physician would not expect to cure dyspepsia, or to cure phthisis, until he had first cured nasal catarrh, if present. I believe that the nails and the teeth are among the best indicators in diagnosis of rheumatism. They show certain signs long before other symptoms appear, except those found in the blood. They may be regarded as an index showing when to cease treatment for rheumatism. When the abrasions on the teeth lose their hypersensitiveness, and the nails lose their corrugations and lines, returning to their normal smoothness and flexibility, we will know that the rheumatic poisons have been eliminated. Can not the ridges so frequently found in the teeth be accounted for through prenatal influences, such as rheumatism during gestation? Why should not the tooth germ become marked as well as the nails? The hardness of enamel retains the ridges, while the nails change about every three months, and the indicator may be lost.

The examination of the blood is important in diagnosis, as this pabulum shows the presence of existing diseases. The examination of the blood is, I think, the strongest basis in diagnosis. In many diseases such evidences may, in their early stages, be found in the blood. These are always found before the objective or subjective symptoms. They are the first to come and the last to leave. Dr. Robert L. Watkins claims that fibrin is present in the blood in advance of the general symptoms of rheumatism, apoplexy, organic heart disease, fevers, etc., and is as variable in form as are the diseases themselves. Dr. Watkins has found in the blood of patients suffering from pyorrhea alveolaris different varieties of fibrin, spores of syphilis, eczema, tubercular matter, and the bacillus itself. He found these even when the diseases were not active. We may, therefore, reasonably base our hope of ascertaining the etiology of the disease by the study of the blood.

Dr. Talbot regards that careless dentistry is the cause for the increase of pyorrhea alveolaris. Dr. Beers touched the key-note when he said: "The gingival margin should not be wounded, greatest care should be taken to heal the wound without infection, especially is this true in cases of inherited tendencies. While the neurotic patient is prone to pyorrhea alveolaris, exaggerated cases are not infrequent in the phlegmatic patient. Although no outward sign of lesion exists, it is often found that the pulps of some teeth involved are devitalized. Infected tissue from septic tooth canals is often classed as pyorrhea alveolaris, and treated without first removing the cause, and from this the teeth are often lost. Whenever such teeth are extracted (in order to prevent the continuation of the disease) the sockets should be curetted and treated as open wounds until filled in by granulation.

Patients naturally shrink from being hurt, but now that we can safely use a strong solution of cocaine, by first administering its antidote, volaseum, the treatment of pyorrhea alveolaris is comparatively painless. I would not have it understood that I believe every tooth involved in this disease can be saved, or that an attempt should be made to save all the teeth, To do the greatest amount of good, it is often necessary to make some sacrifice, but because a number of teeth are loose, it does not follow that they should be extracted. The patient's wishes should be consulted, and as they look to their doctor for guidance, they are generally willing to accept his advice. I was recently asked by a dentist to see with him a case of pyorrhea alveolaris that he had been treating for four years. He appeared satisfied with the results, for by the use of ligatures he had been able to hold in position several loose teeth; yet this was done to the detriment of those teeth to which they were fastened. The gums were hypertrophied, boggy and purple, with evidence of calculi beneath them. Four of the teeth which had not been opened contained septic pulps.

Medical treatment for the dissipation of degenerating causes should be given in conjunction with local treatment, and should be continued until the disease is eradicated—that is, the general health of the patient should receive careful attention. Septic canals should be cleansed and filled with a permanent material. Remove all the calcareous deposits, ulcers, abscess sacs, carious bone, irritating roots, and in fact every source of irritation. The greatest care must be observed in curretting and dissecting away every particle of diseased tissue, so as to leave a fresh and healthy wound, which should be treated as such and encouraged to heal as rapidly as possible. Next, boil out all remaining debris with peroxide of hydrogen. The patient should be instructed to use a sterilizing mouth wash (electrozone is probably the best) every half hour, if possible; then apply tincture of iodine sufficient to flood the wound around the roots. This treatment should be continued every two or three days until the wounds are healed. Care should be observed to avoid blood-letting while treating the wound lest it become infected.

The period of treatment is usually from one to two weeks. The fixation of teeth should be done as early as possible after the first operation by means of a splint, as devised by Dr. Wm. L. Fish. Proper occlusion of the teeth is essential to prevent undue pressue on any one, and to facilitate mastication. Never use acids nor mercury. Operate on a small part at a time. Stop treating the wound when it is healed. When the disease is so extensive that little attachment to the root remains, the pulp being vital, the extirpation of the pulp and the treatment of the canal, if septic, should be done. Instruct the patient to return when any irritation of the gum or jaws is noticed, and every three to six months for examination. It is not always easy to impress upon the patient the importance of this advice. The irritant that caused the disease will reproduce it.—*Dominion Dental Journal.*

